# Genome stability assessment of PRRS vaccine strain with new ARTIC-style sequencing protocol

**DOI:** 10.3389/fvets.2023.1327725

**Published:** 2024-01-08

**Authors:** Szilvia Jakab, Ádám Bálint, Karolina Cseri, Krisztina Bali, Eszter Kaszab, Marianna Domán, Máté Halas, Krisztina Szarka, Krisztián Bányai

**Affiliations:** ^1^Pathogen Discovery Group, HUN-REN Veterinary Medical Research Institute, Budapest, Hungary; ^2^National Laboratory for Infectious Animal Diseases, Antimicrobial Resistance, Veterinary Public Health and Food Chain Safety, Budapest, Hungary; ^3^Veterinary Diagnostic Directorate, National Food Chain Safety Office, Budapest, Hungary; ^4^One Health Institute, University of Debrecen, Debrecen, Hungary; ^5^Department of Metagenomics, University of Debrecen, Debrecen, Hungary; ^6^Prophyl Ltd., Mohács, Hungary; ^7^Department of Pharmacology and Toxicology, University of Veterinary Medicine, Budapest, Hungary

**Keywords:** porcine reproductive and respiratory syndrome virus, Porcilis MLV, genetic variability, single nucleotide variation, deep sequencing, tiling amplicon sequencing

## Abstract

A tiling amplicon sequencing protocol was developed to analyse the genome sequence stability of the modified live PRRSV vaccine strain, Porcilis MLV. The backbone of the ARTIC-style protocol was formed by 34 individual primer pairs, which were divided into two primer pools. Primer pairs were designed to amplify 532 to 588 bp fragments of the corresponding genomic region. The amplicons are suitable for sequencing on Illumina DNA sequencers with available 600-cycle sequencing kits. The concentration of primer pairs in the pools was optimized to obtain a balanced sequencing depth along the genome. Deep sequencing data of three vaccine batches were also analysed. All three vaccine batches were very similar to each other, although they also showed single nucleotide variations (SNVs) affecting less than 1 % of the genome. In the three vaccine strains, 113 to 122 SNV sites were identified; at these sites, the minority variants represented a frequency range of 1 to 48.7 percent. Additionally, the strains within the batches contained well-known length polymorphisms; the genomes of these minority deletion mutants were 135 to 222 bp shorter than the variant with the complete genome. Our results show the usefulness of ARTIC-style protocols in the evaluation of the genomic stability of PRRS MLV strains.

## 1 Introduction

Porcine reproductive and respiratory syndrome (PRRS) remains one of the most devastating viral diseases in pig production systems, responsible for serious economic losses worldwide. The causative viral species, *Betaarterivirus suid 1* and *Betaarterivirus suid 2* (also known as PRRSV-1 and -2, respectively) are enveloped, positive-sense, single-stranded RNA viruses, with a genome of approximately 15 thousand nucleotides (nt) in length (1–3). PRRSV is characterized by high genetic diversity, posing challenges for disease control measures (4, 5).

Modified live virus (MLV) vaccines are widely used tools for the prevention and control of PRRS. These vaccines have been developed to reduce clinical severity and virus shedding, alleviating the disease-associated economic burden. Since their first introduction in 2000, Porcilis^®^ PRRS (MSD Animal Health) vaccines against PRRSV-1 have been preferred vaccines for the active immunisation of sows and growing pigs (6). In general, safety monitoring of vaccines is important during manufacturing, however, information on the genetic stability of vaccine strains in different batches is limited (7, 8). The quasispecies nature of live virus based vaccines poses additional challenges. To date, the whole virus genome sequence of four different Porcilis^®^ PRRS MLV batches has been determined, showing that some deletion variants coexist in different vaccine batches (9). In addition to vaccine stability, another issue to consider is the loss of attenuation of MLV strains in the field that has already been confirmed for some commonly used PRRS MLVs (9–14). Regardless of the theoretic possibility of vaccine strain reversion, data indicate that Porcilis-derived field isolates are genetically more stable, at least based on ORF5 and ORF7 sequence analysis, than some other vaccines (15).

The genetic stability of live vaccines is a critical requirement for their development, production, and field use. Whole genome sequencing on next-generation sequencing platforms permits a more precise description of the population structure of vaccine strains. Tiling amplicon sequencing protocols could overcome sensitivity issues, as next-generation sequencing is preceded by targeted amplification of the genome using a large set of primer pairs. This approach has become very popular since the beginning of the SARS-CoV-2 pandemic. The ARTIC network has developed numerous protocols for direct amplification of virus genomes from clinical samples using tiled, multiplexed primers^1^ (16). In 2020, Gohl and co-workers further developed the approach and established a tailed amplicon-based method for DNA library preparation and NGS sequencing (17). As the target-specific primers can be readily customized, this method can theoretically be adapted on any viral genomes, offering a rapid, sensitive and inexpensive approach to whole genome sequencing.

The aims of this study were the development of a tailed, tiling amplicon sequencing method specific for the Porcilis^®^ PRRS MLV strain and the exploration of the genetic stability of commercial Porcilis^®^ PRRS vaccine batches. This simple, fast, and cost-effective sequencing method could serve as a readily adaptable tool to affirm the quality of PRRS MLVs.

## 2 Materials and methods

### 2.1 Vaccines

Three different batches of Porcilis^®^ PRRS vaccine (batch No. A220CE01, A220AD01, and A221AE01) were used to develop an ARTIC protocol and to compare the genetic stability of the Porcilis MLV strain.

### 2.2 RNA extraction and cDNA synthesis

Vaccine batches were diluted at 1:8 in PBS and then the viral RNA was isolated by the NucleoSpin RNA Virus Kit (Macherey-Nagel, Düren, Germany) according to the manufacturer’s instruction.

Randomly primed reverse transcription was performed using the SuperScript^™^ IV VILO^™^ Master Mix (Thermo Fisher Scientific, Waltham, MA, United States) according to the manufacturer’s instruction. The RT reaction mixture consisted of 4 μL Master Mix (with oligo (dT) 18 and random hexamer primers included), 11 μL nuclease free water and 5 μL RNA template was added. The thermal profile of the reaction was as follows: the denaturation step was performed at 65°C for 5 min, then annealing of primers, the reverse transcription and the inactivation of enzyme lasted 10 min at 25°C, 10 min at 50°C, and 5 min at 85°C, respectively.

### 2.3 Primer design

A tiling amplicon scheme was designed for whole genome amplification. Our aim was to create a robust primer set specific for the Porcilis vaccine strain and suitable for use in multiplex PCRs. Thus, we gathered whole genome records from the GenBank that showed >95% nucleotide sequence identity to the reference strain of Porcilis DV (accession no., KJ127878). This threshold was chosen based on the observation that Porcilis-derived strains display roughly this range of diversity when isolated under field conditions; in this respect, we expected that vaccine production conditions will not permit a high degree of diversification. Whole genome sequences were aligned by the MAFFT algorithm in Geneious Prime^®^ (version 2022.2.2) software and the consensus sequence (threshold: 90%) was extracted and used for primer design. The primer set was designed on the PrimalScheme webserver giving the consensus sequence as input (16).

The amplicon size was adjusted to comply with the 2 × 300 base reading with any Illumina equipment where this option is available. Thus, the expected PCR product size ranged from 532 bp to 588 bp. Afterward, we individually inspected the resulting primer sequences with particular attention to self-, and cross-dimers and we also refined the oligos when needed. The process resulted in 68 primers equivalent to 34 amplicons that theoretically covered the full-length target genome (Table 1). All oligos contained the Illumina-compatible sequencing primer binding site Rd1 SP and Rd2 SP at the 5’ end of the forward and reverse primer, in the following way:

**Table 1:** Features of PRRSV primers tested in this study.

PRRSV specific primer and its sequence		Pool	Position*		Ratio of primers in different pooling schemes		
					v1	v2	v3
1_F	ATGATGTGTAGGGTATTCCCCCTAC	1	1	25	1	3	1.5
1_R	ACTTGGAGTTCACGAAGGTGTC	1	562	583	1	3	1.5
2_F	GCAATCACAACTTCCTCCAACG	2	490	511	1	1	1
2_R	CACGTCGGGGTTYTGGACAA	2	1,015	1,034	1	1	1
3_F	TCTGYCCATTTGAGGAGGCTCA	1	739	760	1	3	3
3_R	AGGCGAACRAATCCTGGGGT	1	1,278	1,297	1	3	3
4_F	CTGTCTTGCCCCCRGTCTTGGATC	2	1,227	1,250	1	1	1
4_R	CTCACCTCTACCTCCCARTGAAC	2	1,710	1,732	1	1	1
5_F	CTTGYTCAGGCGATYCAATGTC	1	1,617	1,638	1	3	1.5
5_R	AARCACYCGTCCAGRGACACA	1	2,147	2,167	1	3	1.5
6_F	ARAAATGCGGTGCCACGGAA	2	2,020	2,039	1	1	1
6_R	GTGTTTGCTCTCTCACAAGGGT	2	2,562	2,583	1	1	1
7_F	CTCRGACYCCATGAAAGRAAAC	1	2,477	2,498	1	3	3
7_R	GCCTCGGTCAATTAAGGCTTG	1	3,030	3,050	1	3	3
8_F	ATGCTCCRGTGGTTGAYGCC	2	2,923	2,942	1	3	3
8_R	TCACTCGACTARGAARATCCGG	2	3,462	3,483	1	3	3
9_F	TGAAGCAACTGGTGGCRCAG	1	3,316	3,335	1	1.5	1.5
9_R	AAAGTTGGCGCTGCTCAAGAG	1	3,868	3,888	1	1.5	1.5
10_F	GTTCTTGGATGGCTTTTGCTGT	2	3,766	3,787	1	1.5	1.5
10_R	CATTTGAYRGCCTGACTGGGAT	2	4,324	4,345	1	1.5	1.5
11_F	ATCAACCRCACCAAAAGCCCAT	1	4,240	4,261	1	1	1
11_R	GACACACAAAGTCGAGAGGAGC	1	4,771	4,792	1	1	1
12_F	TCAAGTGTGTGGCCGAGGAA	2	4,691	4,710	1	1	1
12_R	CAGTTAARGCAGCTCTCCGGAC	2	5,232	5,253	1	1	1
13_F	GACATCCACCAGTACACCTCTG	1	5,154	5,175	1	1	1
13_R	AGTTTGTTTGAACCGGTGTGGA	1	5,692	5,713	1	1	1
14_F	GGAAGGGTTCGCCTTCTGTTTT	2	5,612	5,633	1	1	1
14_R	TGCGTAGAACGCCAGAGAAAGC	2	6,143	6,164	1	1	1
15_F	TCTTTGTGCTTGCATGGGCC	1	6,061	6,080	1	3	1.5
15_R	GCATACGCTGCYTCAATGTACTG	1	6,582	6,604	1	3	1.5
16_F	GTTGTCACAGGCTGACCTTGAT	2	6,494	6,515	1	1	1
16_R	ATCCGTGTAAAAGGTGTCACCG	2	7,040	7,061	1	1	1
17_F	GAGAGGATGAAGAAACAYTGTGT	1	6,957	6,979	1	5	3
17_R	CTCRGAAGTGACTTTTAGGTCTAAAG	1	7,512	7,537	1	5	3
18_F	GGCGGCYTRGTTGTGACTGAAA	2	7,442	7,463	1	5	3
18_R	ATTTACCATCAGACACDGGGGC	2	7,967	7,988	1	5	3
19_F	TRCCTTACAAAACTCCTCAAGACA	1	7,899	7,922	1	1	1
19_R	ACTGAGCGCCGATCTGTGAGC	1	8,453	8,473	1	1	1
20_F	GTCAAGGAGAATTGGCAAACYGT	2	8,348	8,370	1	1	1.5
20_R	ACYAACATAGGCTGAATTTCAAGGA	2	8,892	8,916	1	1	1.5
21_F	ACTGGTAATYTATGCCCAGCAC	1	8,794	8,815	1	1.5	1.5
21_R	TGGCGGAAYTTYTTCCCTTCAT	1	9,324	9,345	1	1.5	1.5
22_F	AGGACCTCATCTGYGGTATTGC	2	9,222	9,243	1	1	1
22_R	CTACTATGAACTTGCTGAGTAGYA	2	9,750	9,773	1	1	1
23_F	ATGGAGACTACCARGTGGTGC	1	9,678	9,698	1	5	3
23_R	ACAGGYCGGGTGGTAAAAAC	1	10,241	10,260	1	5	3
24_F	ACTATTTACAGATTTGGCYCYAACA	2	10,151	10,175	1	1	1
24_R	TRTCYGGGGAAAAGTAAAACCC	2	10,694	10,715	1	1	1
25_F	ACCCRAGGTGCAAGTCTCTCTT	1	10,608	10,629	1	1	1
25_R	AGAGTGCACACGGCTTTAGC	1	11,171	11,190	1	1	1
26_F	GGGGTGTCATCACATYACATCAA	2	11,080	11,102	1	1	1
26_R	CAGTGTAGTCCTTGCCGTCATT	2	11,624	11,645	1	1	1
27_F	CGCAGTGGAAGATTTTGGGGTT	1	11,535	11,556	1	1	1
27_R	GGARACTCGCATGTGCCAAA	1	12,079	12,098	1	1	1
28_F	ACTATCGAAGGTCCTATGAARGCTT	2	11,995	12,019	1	1	1
28_R	TGATGGTYAGCTCGAATGATGT	2	12,530	12,551	1	1	1
29_F	TGTGGCTTCATCTGTTACCTTGT	1	12,440	12,462	1	1	1.5
29_R	CGAACGCCTCAGAAACCATGA	1	12,989	13,009	1	1	1.5
30_F	AATAGACGGGGGCAATTGGTTC	2	12,916	12,937	1	1	1
30_R	CAATGGTTGTAGCCCACCTCAT	2	13,444	13,465	1	1	1
31_F	CCAACATACCCAGCAGCATCAT	1	13,374	13,395	1	1	1
31_R	TGAAGTTGGTAAACCGGGTACG	1	13,911	13,932	1	1	1
32_F	CTTTCGCAGCGYTCGTATGTT	2	13,846	13,866	1	1	1
32_R	GGCAACACAATCTGCATCTGGA	2	14,372	14,393	1	1	1
33_F	CACCAACCGTGTCGCAYTTAC	1	14,284	14,304	1	1	1
33_R	CGTCTGGATCGATTGCAAGCAG	1	14,822	14,843	1	1	1
34_F	TCTAGTACCAGGACTTCGGAGC	2	14,521	14,542	1	1	1
34_R	TTAATTTCGGTCACATGGTTCCTGC	2	15,076	15,100	1	1	1

*Relative to reference genome KF991509.

forward: TCGTCGGCAGCGTCAGATGTGTATAAGAGACAG <PRRSV specific primer>

and reverse: GTCTCGTGGGCTCGGAGATGTGTATAAGAGACAG <PRRSV specific primer>. Illumina-compatible forward and reverse indexing primers contained *part of the sequencing primer binding sites*, **i5 and i7 indices** (Nextera XT Index Kit v2 Set C) and the P5 and P7 adapters, respectively.

(F: 5’-AATGATACGGCGACCACCGAGATCTACAC**i5***TCGTCGGCAGCGTC*-3’,

R: 5’-CAAGCAGAAGACGGCATACGAGAT**i7***GTCTCGTGGGCTCGG*-3’).

To generate the primer pools, primers were resuspended to a stock concentration of 100 μM and then divided into two pools (poolAv1, from pool A1 and A2, respectively) by combining the same volume of odd and even numbered amplicons. Two other pooling schemes, poolAv2 and poolAv3, were prepared to adjust the relative proportions of primers, and consequently amplicons, to achieve a more balanced coverage of the genome.

### 2.4 Library preparation and next generation sequencing

Whole genome amplification connected with library preparation consisted of a two-step PCR protocol. Both PCR rounds (PCR-1 and PCR-2) were performed using Q5U^®^ Hot Start High-Fidelity DNA Polymerase (NEB, Frankfurt, Germany) according to the manufacturer’s instruction. Separate PCR-1 reactions for the multiplex primer set of pool A1 and A2 were composed of 15.46 μL nuclease-free water, 5 μL 5X Q5U Reaction Buffer, 0.5 μL dNTP Mix (10 mM), 1.29 μL primer pool A1 or A2 (10 μM, final concentration of 0.015 μM per primer), 0.25 μL Q5U Hot Start High-Fidelity DNA Polymerase and 2.5 μL cDNA template. PCR cycling was performed at 98°C for 30 s, followed by 30 cycles of 98°C for 15 s and 65°C for 5 min, then held at 4°C. PCR-1 products were analysed on 1% agarose gel, the bands were excised at 600 bp, or so, and the DNA were purified from gel slices with the Gel/PCR DNA Fragments Extraction Kit (Favorgen, Ping Tung, Taiwan). After gel extraction, pool A1 and A2 were combined then diluted 1:100 for each sample. During the second PCR, Illumina specific adapters and indices were added to the PCR-1 products. PCR-2 composed of 11.75 μL nuclease-free water, 5 μL 5X Q5U Reaction Buffer, 0.5 μL dNTP Mix (10 mM), 1.25–1.25 μL forward and reverse indexing primers (10 μM), 0.25 μL Q5U Hot Start High-Fidelity DNA Polymerase and 5 μL pooled and diluted DNA template of PCR-1. PCR cycling was performed at 98°C for 30 s, 30 cycles of 98°C for 20 s, 55°C for 15 s and 72°C for 1 min, the final extension lasted for 5 min at 72°C, then held at 4°C. Again, PCR-2 products were analysed on 1% agarose gel, the bands were excised at ~600 bp, and the gel slices were isolated with Gel/PCR DNA Fragments Extraction Kit (Favorgen, Ping Tung, Taiwan).

The concentrations of DNA libraries were measured using a Qubit equipment and then diluted to 4 nM. This pool was denatured and then diluted to 20 pM, spiked with 35% PhiX, and sequenced on an Illumina Miseq sequencer using the Reagent Kit v3 (600-cycle) (Illumina, San Diego, CA, United States).

### 2.5 Sequence data analysis

First, the Illumina sequence reads were trimmed as follows: sequence reads under 75 bp were discarded, the PRRSV specific primer sequences and low-quality bases (minimum Phred score of 10) were removed at both ends using BBDuk plugin in Geneious Prime^®^. Second, genome assembly was performed in Geneious Prime^®^ with the built-in Geneious algorithm, or if necessary, with the Minimap2 assembler, implementing reference mapping to a Porcilis DV strain (accession no., KF991509).

Intra-strain sequence variability was analysed from the short-read data of poolAv3. For the analysis of single nucleotide variants (SNVs) the sequence reads under 75 bp were discarded, the PRRSV specific primer sequences and low-quality bases (minimum Phred score of 30) were removed at both ends using BBDuk plugin in Geneious Prime^®^. We used the built-in algorithm of Geneious Prime^®^ for variant calling with minimum variant frequency of 1%. Only SNVs that were identified on parallel sequencing runs were accepted.

## 3 Results and discussion

### 3.1 Assay evaluation

In this study, we developed and tested a tiling amplicon sequencing protocol specific for a widely used PRRSV vaccine strain, Porcilis MLV. The workflow was based on a two-step PCR library preparation method. We first examined whether the designed primer set successfully amplifies different batches of Porcilis MLV. In this respect, we encountered an immediate obstacle, as the study was launched after Hungary was declared PRRS-free and the Porcilis MLV vaccine was available in limited quantities on the market. Next to the evaluation of individual primer combinations, we focused on the improvement of primer balance in individual primer pools to achieve better sequence coverage across the genome.

**Figure 1:**
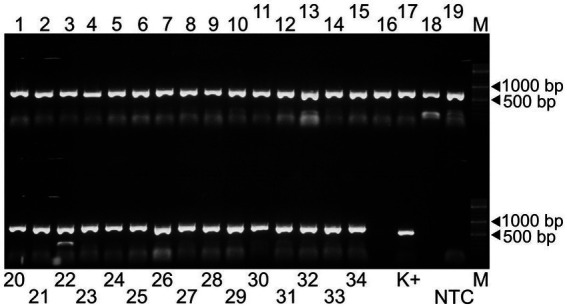
Agarose gel electrophoresis of the 34 amplicons generated by the Porcilis MLV specific primer pairs (lanes 1 to 34). The PCR product obtained by a diagnostic primer pair targeting the ORF7 region was used as positive control (K+) (18). NTC and M refer to the non-template control and the GeneRuler DNA Ladder Mix, respectively.

**Figure 2:**
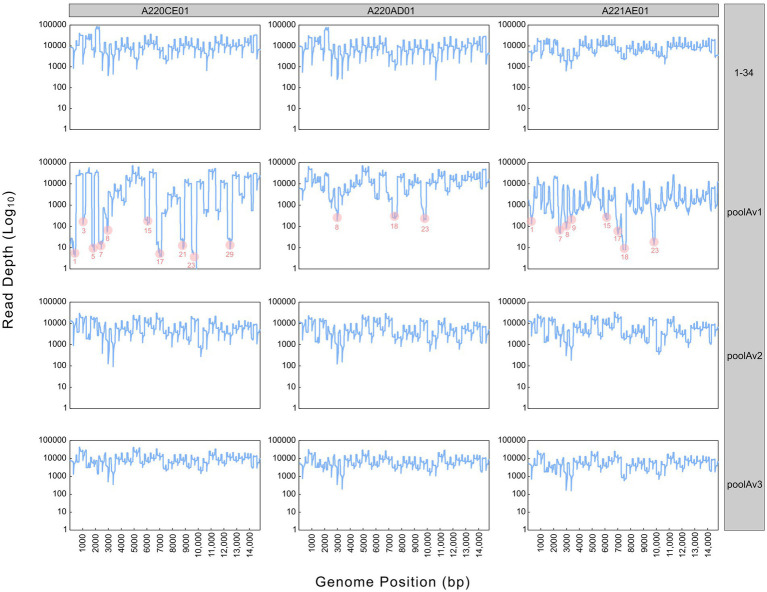
Sequencing depth derived from different pooling approaches (1–34, poolAv1, poolAv2, poolAv3) as determined for the three vaccine batches (A220CE01, A220AD01, A221AE01). In the plots linked to poolAv1, the drop-off amplicons are highlighted.

In the first step of the test development, our results showed that all 34 primer pairs generated PCR products of the expected size in the separate reactions (Figure 1). These PCR products were then pooled to obtain equimolar ratios for each amplicon (1 to 34) and then subjected these pools to sequencing. The overall sequencing depth ranged from 9,251x to 13,889x and the assembled contig covered the full-length genome (Figure 2). These data indicated that the assay design met our expectations. However, because it was not practical to use the primer pairs in separate reaction tubes, the primers were pooled. In this practice, we followed the protocols previously established for other viruses and combined the even and odd primer pairs in separate reaction tubes (poolAv1) (16, 17). Thus, two primer pool mixtures were prepared and the cDNA templates were amplified under same conditions in these two reaction tubes during the PCR-1 round.

Sequencing the Porcilis vaccine strain from different batches was successful with poolAv1 (Figure 2). Genome coverage, depending on the vaccine batch, approached or reached 100%, while sequencing depth varied within and between all vaccine batches. We observed a reduction of the sequencing depth at amplicons 1, 3, 5, 7, 8, 15, 17, 21, 23, and 29 for A220CE01, at amplicons 8, 18 and 23 for A220AD01, and at amplicons 1, 7, 8, 9, 15, 17, 18, and 23 for A221AE01, respectively (Figure 2). Thus, 7 out of 12 primer pairs resulted in reduced amount of their respective PCR products in at least two distinct vaccine batches. Moreover, in case of amplicon 23, no reads were generated in A220CE01. To better evaluate the efficiency of the primer pairs, we calculated the mean sequencing depth per amplicon and per vaccine sample by excluding overlapping amplicon regions. The mean sequencing depth was 9,944x, 11,069x, and 1799x, whereas the range of sequencing depths ranged between 3x and 39,624x, 290x and 37,921x, and 12x and 18,107x for A220CE01, A220AD01, and A221AE01, respectively. The varying efficiencies of primer pairs used in equimolar ratio prompted us to optimize the assay.

To improve the primer balance we modified the working concentration of selected primers in the reaction mixtures and prepared two distinct experimental primer concentrations (Table 1). As such, we prepared two other variations of poolA, designated as v2 and v3. Figure 2 shows the coverage and sequencing depth achieved by using primer pools with different concentration ratios of individual primers. By using poolAv2 and poolAv3, we reached a more balanced sequencing depth along the entire genome, compared to the poolAv1. For example, the differences between the minimum and maximum depth per amplicon achieved by poolAv3 for the three vaccine batches was 14- to 16-fold compared to the 131- to 14,151-fold difference observed when primers were used in equimolar ratios (poolAv1). Overall, the primer concentration optimization resulted in a significant decrease of the number of low depth regions and simultaneously diminished regions without sequence reads. As a result, we achieved a more balanced sequencing depth for the entire genome. The need to optimize primer concentrations was already demonstrated for other tiling amplicon based whole genome sequencing protocols, for example, for the widely used SARS-CoV-2 ARTIC primers (19).

### 3.2 Vaccine strain variability

When comparing consensus sequences generated by the four different pooling approaches, such as the separate PCR-1 with 34 primer pairs and poolA with the three different pooling schemes, we identified some nucleotide differences in the assembled consensus genome sequences. In the consensus genome of A220CE01 from poolAv1 and 1–34, in A220AD01 from poolAv2, and in A221AE01 from poolAv1 we identified, respectively, 6 nt and 2 nt, 1 nt, and 5 nt differences from the others of the given batches. Among these, 1 (A220CE01), 1 (A220AD01), and 3 (A221AE01) mutations corresponded to the identified SNV sites. To further explore the vaccine strain variation, short-read sequences generated by the poolAv3 were used for genomic analyses.

Previously, three different deletion variants of the Porcilis strain were found in some vaccine batches; these were designated as LONG-DEL, SHORT-DEL, and SHIFT-DEL. The difference among the three variants were seen in the genomic regions 2,216 to 2,437 for LONG-DEL, 2,231 to 2,365 for SHORT-DEL, 2,344 to 2,435 and 2,446 to 2,506 for SHIFT-DEL (9). When analysing data from poolAv3, beside the FULL-LENGTH variant, we could also detect all deletion variants in the three vaccine batches, and we estimated that the LONG-DEL variant was the most abundant form of the Porcilis vaccine strain, a finding that corresponds to previously published results (9). Downstream genetic analyses were conducted with the LONG-DEL variant. In brief, all three assembled LONG-DEL genomic variants of the Porcilis vaccine strain were 14,853 nt long.

Sites 1_F (5’-ATGATGTGTAGGGTATTCCCCCCCCCCCTAC-3’) and 34_R (5’-TTAATTTCGGTCACATGGTTCCTGC-3’) at the 5’ and 3’ ends of the genome, respectively, were excluded from the assembly, as no overlapping amplicons were generated in this region.

**Figure 3:**
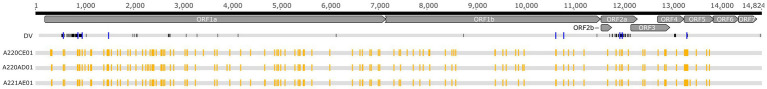
Schematic alignment of the Porcilis DV strain and the vaccine batches investigated in our study. Yellow squares show the SNV distribution within the vaccine batches whereas blue squares display SNV sites that correspond to the nucleotide differences between the DV and the vaccine batches.

The complete genomes derived from the three vaccine batches were nearly identical, only one polymorph site was detected at position 1,519 (the position is relative to the Porcilis DV strain; accession no., KF991509). The pairwise nucleotide identity of all available Porcilis complete genomes from different vaccine batches, including the strains of this study, varied between 99.9 and 100% (the deleted region at nt position 2,215–2,506 was excluded from the alignment and the calculations). When the vaccine strains were compared to the wild-type DV strain (accession no., KJ127878) the nucleotide identity values were as high as 99.1%. In fact, we observed a total of 126 nt difference between vaccine strains and the wild-type DV strain. These mutations accumulated chiefly within the 5’ end of the ORF1 region and the ORF2a region (Figure 3).

Based on the analyses of amplicon deep sequencing, we detected high genetic complexity. Our analyses of intra-strain variability showed that 122, 115, and 113 SNV positions were present in the Porcilis PRRS batches of A220CE01, A220AD01, and A221AE01, respectively (Figure 3). Most of the minor variants (a total of 104 sites) in these assemblies were identical in all three vaccine batches. The mean frequency of SNV bases in the minor variants, calculated from duplicated amplicon libraries, varied between 1.1 to 48.7%, 1 to 47.2%, and 1.3 to 48.1%, respectively, for the three batches (Supplementary Figure S1). The majority of SNVs was found in the ORF1a of all three genomes (55.4 to 60.9%), while no SNVs were detected at all in the ORF6 and ORF7 regions. The number of SNVs across the whole genome found in our vaccine batches was much lower than in some field strains, including a PRRSV-2 MLV-like strain (20, 21). Altogether we observed a maximum of 15 nt difference between our vaccine batches and previously sequenced Porcilis strains, and 7 of these differences were identified as minor variants in our SNV analysis, suggesting that the quasispecies structure partly contributes to the identified inter-vaccine-vial diversity (Supplementary Figure S2).

The intra-strain variability of the Porcilis MLV vaccine was determined directly from vaccine vials. According to previous studies the uneven distribution and the accumulation of SNVs in the ORF1a region is very typical and it is irrelevant whether the study strain is a vaccine derivative, or, a wild-type PRRSV strain (20–22). The absence of SNVs in the ORF6 and ORF7 regions was not unexpected, as these coding regions are highly conserved, even though some field strains harbour measurable mutant spectra at these sites (20–23). Importantly, the pattern of SNVs in the Porcilis vaccine strains did not entirely correspond with sites that differ between the DV strain these sites; a total of 9 minor variants were shared between them, six in ORF1a, two in ORF2a and one in ORF5 (Figure 3).

## 4 Conclusion

ARTIC-style protocols suitable for the amplification of whole viral genomes have become a keystone for some years to describe genetic diversity and to support molecular epidemiological investigations. In our study, we developed and evaluated an ARTIC-style sequencing method for PRRSV. Our assay was designed to be able to assess the genetic stability of vaccine strains of Porcilis MLV, but we anticipate that the assay can be readily adapted to other PRRSV MLVs. Further efforts are needed to demonstrate the potential field application of this protocol, as frequent recombination events between vaccine and wild-type PRRSVs in the field may pose a challenge to the successful design and implementation of broad-range ARTIC methods. Such leaps in viral genome evolution could erroneously undermine the value of these assays if common evolutionary mechanisms are not taken into account when evaluating the obtained sequence data.

## Data availability statement

The datasets presented in this study can be found in online repositories. The names of the repository/repositories and accession number(s) can be found at: https://www.ncbi.nlm.nih.gov/genbank/, PRJNA1026488.

## Author contributions

SJ: Formal analysis, Methodology, Writing – original draft, Writing – review & editing. ÁB: Resources, Supervision, Writing – review & editing. KC: Methodology, Writing – review & editing. KBl: Formal analysis, Methodology, Writing – review & editing. EK: Formal analysis, Methodology, Writing – review & editing. MD: Methodology, Validation, Writing – review & editing. MH: Resources, Supervision, Writing – review & editing. KS: Methodology, Supervision, Writing – review & editing. KBn: Conceptualization, Funding acquisition, Supervision, Writing – review & editing.

## Footnotes

^1^ https://artic.network/
