# Supplementary material for: Genome stability assessment of PRRS vaccine strain with new ARTIC-style sequencing protocol
Source: Front Vet Sci. 2024 Jan 8;10:1327725. doi: 10.3389/fvets.2023.1327725 (PMC10800885; doi:10.3389/fvets.2023.1327725)
Supplement: Supplementary file 1 [file Data_Sheet_1.docx]

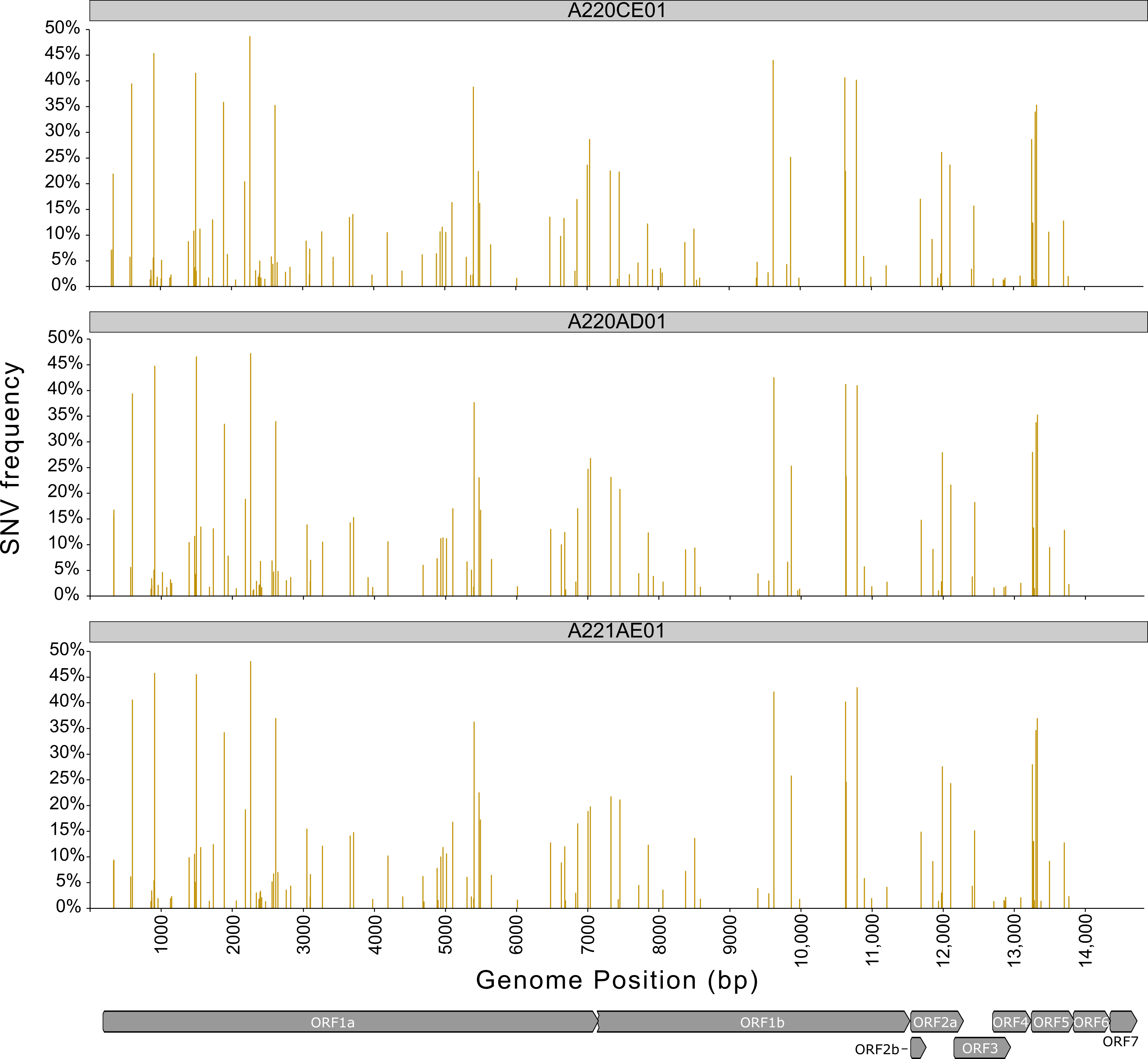
**Supplementary Figure 1**. Distribution and frequency of the identified SNV sites along the complete genomes of the vaccine batches.


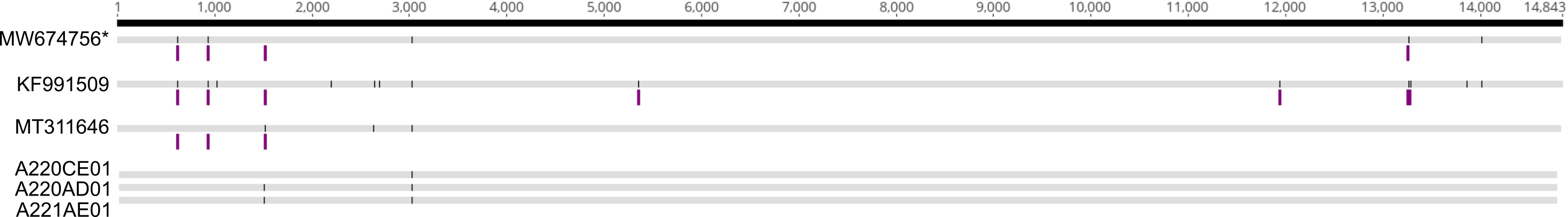
**Supplementary Figure 2**. Alignment of all available Porcilis MLV complete genomes. Black lines indicate nt differences in the alignment, and the purple squares correspond to sites that were identical to a detected SNV within the vaccine batches in our study.

* Two different vaccine batches had the same complete genome according to Eclercy et al., 2021.
